# Geriatric nutritional risk index and adverse medical outcomes among Egyptian patients admitted to a geriatric hospital: a prospective cohort study

**DOI:** 10.1186/s12877-024-04671-5

**Published:** 2024-01-15

**Authors:** Hebatullah O Mohammed, Azza M. Hassan, Aya Mostafa, Mohamed S. Khater, Aisha Aboelfotoh, Khaled M. Abd Elaziz

**Affiliations:** 1 Department of community, environmental and occupational medicine. Faculty of medicine, Ain Shams University, Cairo, 11566 Egypt; 2 Department of geriatrics and gerontology. Faculty of medicine, Ain Shams University, Cairo, 11566 Egypt

**Keywords:** Geriatric, Nutritional risk, Length of stay, Malnutrition, Mortality

## Abstract

**Background:**

Elderly are one of the most heterogeneous and vulnerable groups who have a higher risk of nutritional problems. Malnutrition is prevalent among hospitalized elderly but underdiagnosed and almost undistinguishable from the changes in the aging process. The Geriatric Nutritional Risk Index (GNRI) is a tool created to predict nutrition-related complications in hospitalized patients. This study aims to measure the prevalence of nutritional risk using the GNRI among hospitalized elderly Egyptian inpatients and to determine the association between the GNRI and selected adverse clinical outcomes.

**Methods:**

A hospital-based prospective cohort study was conducted among 334 elderly patients admitted to a tertiary specialized geriatric university hospital in Cairo, Egypt from August 2021 to June 2022. Within 48 hours after hospital admission, socio-demographic characteristics, blood biomarkers, anthropometric measurements, and nutritional risk assessment by the GNRI score were obtained. Patients were divided into three groups based on their GNRI: high, low, and no nutritional risk (GNRI<92, 92-98, and >98) respectively. Patients were followed up for the occurrence of adverse outcomes during hospital stay (bed sores, Healthcare-Associated Infections (HAIs), hospital Length of Stay (LOS), and hospital mortality) and three months after discharge (non-improvement medical status, appearance of new medical conditions, hospital readmission and 90-day mortality). Multivariable regression and survival analysis were conducted.

**Results:**

The prevalence of high-nutritional risk was 45.5% (95% CI, 40%–51%). Patients with high risk had significantly longer LOS than those with no risk. The high-nutritional risk was significantly associated with the development of bed sores (Adjusted Odds Ratio (AOR) 4.89; 95% CI, 1.37–17.45), HAIs (AOR: 3.18; 95% CI, 1.48–6.83), and hospital mortality (AOR: 4.41; 95% CI, 1.04–18.59). The overall survival rate was significantly lower among patients with high-nutritional risk compared to those with no risk.

**Conclusion:**

GNRI is a simple and easily applicable objective nutritional screening tool with high prognostic value in this Egyptian sample of patients. The findings of this study signal the initiation of the application of this tool to all geriatric hospitals in Egypt.

## Introduction

Nutritional status is often compromised in the elderly. Physiological and social changes resulting from advanced age, comorbidities, high consumption of drugs, degenerative loss of mobility, psychological and mental distress, and loss of appetite are just some of the factors that affect the nutritional status of this age group [1, 2].

Hospitalized elderly patients have the highest risk of being at nutritional risk or becoming malnourished. During hospitalization, multiple factors such as underlying acute or chronic diseases, inflammatory states, and infections increase patients' energy expenditure while reducing their normal nutrient intake [3].

The consequences of malnutrition in hospitalized elderly result in multiple adverse outcomes such as increased prevalence of Healthcare-Associated Infections (HAIs), decreased functional status, decreased quality of life, longer hospital Length of Stay (LOS), increased healthcare costs, hospital readmission rate, and hospital mortality [4].

Malnutrition and nutritional risk are common in hospitalized elderly. But unfortunately, is not easily recognizable or distinguishable from the changes in the aging process, which means that a significant percentage of patients are undiagnosed [5]. The prevalence of malnutrition among the elderly in hospital settings ranges from 11% to 55% internationally [6]. A hospital-based cross-sectional study was carried out in the medical Intensive Care Unit (ICU) of the internal medicine ward in AL-Zahra University Hospital, Cairo, Egypt. By nutritional assessment, (50%) of patients were malnourished either mild/moderate (35.3%) or severely malnourished (14.7%) [7]. Another study carried out at Zagazig University Hospitals, Egypt reported that (51.5%) of the studied elderly were at risk for malnutrition [8].

Malnutrition underdiagnosis can be prevented, possibly reducing the prevalence of malnourished hospitalized elderly patients. This happens using various nutritional screening tools which become an essential step to classify those patients who are at nutritional risk from hundreds of patients attending tertiary care hospitals, especially in developing countries like Egypt. Then intervene immediately by developing appropriate nutritional care plans that could improve their prognosis [9].

There are many tools for nutritional screening and identifying nutritional risks in the elderly population. Among the validated measures, are the Malnutrition Inflammation Score (MIS) and the Subjective Global Assessment (SGA). Both are based on medical history and clinical findings, and they need subjective assessment and judgment by the highly trained examiner to verify consistent results among different examiners and at different times [10].

Other nutritional screening tools include Mini Nutritional Assessment–Short Form (MNA-SF) [11], Malnutrition Universal Screening Tool (MUST) [12], Malnutrition Screening Tool (MST) [13], and Nutritional Risk Screening 2002 (NRS-2002) [14]. Although the method recommended by the European Society of Parenteral and Enteral Nutrition (ESPEN) for assessing the nutritional status of older people is the Mini Nutritional Assessment (MNA) [15]. But it does not apply to those patients diagnosed with dementia or other communication problems [16]. Subjective data about the history of weight loss and calculations of the weight loss percentage in MUST, NRS-2002, and MST may be a barrier as they rely on memory and take more time for the busy healthcare staff on the wards [17].

The Geriatric Nutrition Risk Index (GNRI) is a simple and objective screening index designed specifically for the hospitalized elderly to assess nutritional risk and predict nutrition-related complications [18]. It allows clinicians to assess patients easily based on two main parameters: serum albumin and the ratio between the current and ideal weight of the individual. It was developed in response to the fact that elderly patients are often unable to participate in questionnaire‐based assessments as used in MNA. Also, it did not depend on a caregiver or memory. Therefore, it is practical and provides reliable assessment in most healthcare settings, especially among elderly patients who have cognitive impairment or delirium and dementia [9].

A cross-sectional study was conducted in the Geriatrics and Gerontology Department at Ain Shams University Hospital in Cairo Egypt to compare the performance and the accuracy of different nutritional screening tools. It reported that among the several studied assessment tools, NRS-2002 had the highest sensitivity while GNRI had the highest specificity [19]. Another study was carried out at Alexandria Main University Hospital, and the prevalence of risk of malnutrition among a sample of elderly patients aged ≥65 years as assessed by GNRI was (33.3%) [20].

Although GNRI has been validated by more than one study, only a few studies were conducted in Egypt, and none studies the role of GNRI in the prediction of nutrition-related complications and mortality after discharge among the elderly population.

Thus, this study aimed primarily to investigate whether nutritional risk, as assessed by the GNRI, is associated with multiple adverse outcomes in elderly patients admitted to the geriatric hospital Ain Shams University. Secondly to study the capability of the GNRI to predict adverse outcomes and mortality during hospitalization and up to 90 days after discharge.

## Subjects & methods

### Study design and population

This hospital-based prospective cohort study was conducted in the Geriatric Hospital at Ain Shams University, Cairo, Egypt from August 2021 to June 2022. Eligible patients were aged ≥ 60 years and had an anticipated length of stay of at least 48 hours. Exclusion criteria were: (i) presence of well-known liver, renal or neoplastic disorders, (ii) Haemodialysis patient, (iii) Severe swelling affecting body weight (such as asities, decompensated heart failure, generalized edema, and elephantiasis), (iv) Amputation of the lower limb, hemiplegia, and paraplegia, and (v) terminal ill condition (ICU patients).

### Sample size and technique

Using Epi info program version 7 for sample size calculation, setting the confidence interval at 95% and margin of error at 5%, it is estimated that a sample size of 334 patients was enough to detect an expected prevalence of nutritional risk of 68% [18].

All eligible elderly patients admitted to the internal ward of the Geriatric hospital Ain Shams University were consecutively enrolled until the sample size was obtained.

### Data collection

#### Data extraction sheet

All patients were assessed within 48 hours of admission. The demographic characteristics that were collected included age, gender, level of education, marital status, income, and presence of a caregiver. Patient clinical information and associated comorbidities were also collected.

#### Nutritional assessment

1. Anthropometric measurements
  The following anthropometric nutritional parameters: actual (present) weight, height, Body Mass Index (BMI) (in kg/m2), Triceps skinfold thickness, Mid-Arm Circumference (MAC), and Calf Circumference (CC) were obtained.
  Weight was determined on a calibrated scale placed on a hard-floor surface. Participants had to be in light clothing and without shoes, and measurements were recorded to the nearest 0.5 kg. Standing height was measured using a tape measure, the patients stood up straight with heels together and height was recorded to the nearest 0.5 cm. In the case of bedridden Estimated height (EH) was extrapolated from Knee-Heel (KH) length according to the equations [21]:
  $$\mathrm{For men}:\mathrm{ H }({\text{cm}}) = [2.02 *\mathrm{ KH }({\text{cm}})] [0.04 *\mathrm{ age }({\text{y}})] + 64.19$$
  $$\mathrm{or women}:\mathrm{ H }({\text{cm}}) = [1.83 *\mathrm{KH }({\text{cm}})] [0.24 *\mathrm{ age }({\text{y}})] + 84.88$$
  BMI was calculated as weight (in kg) divided by height squared (by m2). MAC was measured by asking the patient to bend his non-dominant arm at the elbow at a right angle with the palm up; then, the distance between the acromial surfaces of the scapula and the olecranon process of the elbow was measured and the tape at the mid-point on the upper arm tightened snugly. MAC was recorded to the nearest 0.1 cm. Triceps skinfold thickness by a skinfold caliper. CC was measured by asking the patient to sit with the left leg hanging loosely, wrapping the tape around the calf at the widest part, and noting the measurement. CC was recorded to the nearest 0.1 cm [22].
2. Blood biomarkers levels
  Laboratory assessments done were serum levels of albumin (g/dL), total protein (g/dL), hemoglobin (g/dL), C reactive protein (mg/L), and ferritin (ng/mL). All these investigations were done to patients within 48 hours after hospital admission.
3. Geriatric Nutrition Risk Index (GNRI)
  The nutrition-related risk was evaluated using the GNRI within 48 hours of admission.
  It was calculated as follows [23]:
  $$\mathrm{GNRI }= [1.489\hspace{0.17em}\times \hspace{0.17em}\mathrm{serum albumin }({\text{g}}/{\text{L}})]\hspace{0.17em}+\hspace{0.17em}[41.7\hspace{0.17em}\times \hspace{0.17em}\mathrm{present weight}/\mathrm{ideal weight }({\text{kg}})]$$
  Ideal body weight was derived using the following equations of Lorentz (WLo) [23]:
  $$\text{ideal weight for men} = \text{heigth (cm)} - 100 [(\text{height} - 150)/4]$$
  $$\text{ideal weight for women} = \text{heigth (cm)} - 100 [(\text{height} - 150)/2.5]$$
  Study participants were categorized into the following three categories: no nutritional risk (GNRI >98), low nutritional risk (92–98), and high nutritional risk (GNRI <92).
  In total, 356 hospitalized elderly patients who were admitted to the geriatric hospital Ain shams university were assessed, of whom 22 were excluded due to the presence of exclusion criteria.

### Outcomes

Patients were followed starting from the date of assessment, during the hospital stay, and for three months after discharge for the occurrence of selected clinical complications. The primary adverse outcomes that may occur at the hospital were bed sores, HAIs, hospital-acquired Coronavirus disease 2019 (COVID-19) infection, prolonged hospital LOS, and hospital mortality (primary endpoint). HAIs are infection(s) acquired during the process of receiving health care that was not present during the time of admission, such as urinary tract infection, pneumonia, surgical site infection, and bloodstream infection [24]. Hospital LOS is defined as the actual number of days in the hospital from the day of admission to the day of discharge or death (if death occurred in the hospital) [25]. It was obtained from hospital charts. The secondary outcomes that occurred after discharge were non-improvement in the medical status, appearance of new medical conditions, hospital readmission, and 90-day mortality (secondary endpoint).

### Data management and statistical analysis

The collected data were revised for completeness, coded, and entered into a personal computer. All data manipulation and statistical analyses were performed using IBM SPSS (Statistical Package for Social Science) software version 24.0. Qualitative categorical variables were expressed as frequencies and percentages. Quantitative variables were expressed as means with the Standard Deviation (SD). One-way Analysis of Variance (ANOVA), Kruskal–Wallis, and Chi-square tests were used. Multivariable logistic regression analyses were performed with GNRI as the independent variable (with GNRI >98, normal nutritional status, as the reference group). Bed sores, HAIs, hospital mortality, post-discharge health complications, and hospital readmission were the dependent variables. Overall Survival (OS) curves were plotted using the Kaplan–Meier method and compared using the generalized log-rank test. The Cox proportional hazards model was conducted to determine the independent predictors of overall mortality in the study participants. Adjusted Hazard Ratios (AHRs) and 95% confidence intervals (CIs) were reported. *P* ≤ 0.05 was considered statistically significant.

## Results

The total number of elderly hospitalized patients included in this study is 334.

The baseline demographic and clinical characteristics of the patients according to GNRI are provided in Table 1. The mean age of these patients was 72.35 + 8.1 years and (55.7%) were females. Regarding preadmission status, about half of the patients (51.5%) had no priorly admission and came from home and (44%) were in geriatric hospital ICU and then transferred to hospital wards. The patients with lower GNRI levels had a significantly greater mean age. However, there were no statistically significant differences in gender, education, marital status, presence of a caregiver, and income among nutritional risk categories**.** Lower GNRI levels were significantly associated with lower serum albumin levels, total Protein, haemoglobin, BMI, triceps skin fold thickness, MAC, and CC. On the other hand, the levels of CRP and Ferritin were significantly higher in the high-risk group than no-risk (Table 1).

**Table 1:** Baseline patients characteristics according to geriatric nutritional risk index levels

**Patients’ characteristics**		**Total patients (*n*=334)**	**High nutritional risk (*n*=152)**	**Low nutritional risk (*n*=60)**	**No nutritional risk (*n*=122)**	***P*-value **^**a**^
**Age**	**Mean ±SD**	72.35 ±8.1	73.7 ± 8.5	73.6 ± 7.8	69.9 ±7 ^b^	**<0.001**
		**No. (%)**				
**Gender**	**Male**	148 (44.3)	78 (51.3)	23 (38.3)	47 (38.5)	0.062
	**Female**	186 (55.7)	74 (48.7)	37 (61.7)	75 (61.5)	
**Education status**	**Illiterate**	175 (52.4)	87 (57.2)	29 (48.3)	59 (48.4)	0.196
	**Read &write**	37 (11.0)	17 (11.2)	10 (16.7)	10 (8.2)	
	**Primary**	42 (12.6)	11 (7.2)	11 (18.3)	20 (16.4)	
	**Preparatory**	14 (4.2)	7 (4.6)	1 (1.7)	6 (4.9)	
	**Secondary (Diploma)**	40 (12.0)	17 (11.2)	7 (11.7)	16 (13.1)	
	**University (higher institute) / or above**	26 (7.8)	13 (8.6)	2 (3.3)	11 (9.0)	
**Marital status**	**Married**	152 (45.5)	70 (46.1)	25 (41.7)	57 (46.7)	0.906
	**Widowed**	167 (50.0)	75 (49.3)	33 (55.0)	59 (48.4)	
	**Divorced**	9 (2.7)	4 (2.6)	2 (3.3)	3 (2.5)	
	**Single**	6 (1.8)	3 (2.0)	0 (0.0)	3 (2.5)	
**Care giver**	**partner**	148 (44.3)	68 (44.7)	24 (40.0)	56 (45.9)	0.546
	**Own Family**	125 (37.4)	59 (38.8)	24 (40.0)	42 (34.4)	
	**Relatives**	35 (10.5)	18 (11.8)	6 (10.0)	11 (9.0)	
	**live alone**	26 (7.8)	7 (4.6)	6 (10.0)	13 (10.7)	
**Family income**	**Salary**	18 (5.4)	9 (5.9)	0 (0.0)	9 (7.4)	0.343
	**sufficient pension**	83 (24.8)	36 (23.7)	18 (30.0)	29 (23.8)	
	**not sufficient pension**	177 (53.0)	77 (50.7)	33 (55.0)	67 (54.9)	
	**Social support**	56 (16.8)	30 (19.7)	9 (15.0)	17 (13.9)	
**The status prior to admission**	**In another hospital**	9 (2.7)	5 (3.3)	1 (1.7)	3 (2.5)	**0.008**
	**another department in ASU hospital**	6 (1.8)	5 (3.3)	0 (0.0)	1 (0.8)	
	**In Intensive Care Unit**	147 (44.0)	80 (52.6)	27 (45.0)	40 (32.8)	
	**At home**	172 (51.5)	62 (40.8)	32 (53.3)	78 (63.9)	
**Laboratory investigation**	**Serum Albumin (g/dL)**	3.1 ± 0.6	2.7 ± 0.5^b^	3.1 ± 0.4^b^	3.5 ± 0.5^b^	**<0.001**
	**Total Protein (g/dL)**	6.2 ± 0.8	5.8 ± 0.8^b^	6.3 ± 0.6	6.6 ± 0.8	**0.005**
	**Haemoglobin (g/dL)**	10.5 ± 4.5	9.7 ± 1.7^b^	10.6 ± 1.9	11.6 ± 7.1	**<0.001**
	**C reactive protein (mg/L)**	34.9 (57.3)	46.8 (63.2)^b^	23.5 (93.2) ^b^	25.0 (78.1) ^b^	**0.007**
	**Ferritin (ng/mL)**	334.2 (724.6)	757(1128.5) ^b^	334.2 (1288.2) ^b^	155.7 (419.1) ^b^	**<0.001**
**Anthropometric measures**	**BMI**	26.32 ± 4.9	23.55 ± 3.2^b^	26.02 ± 2.8^b^	29.92 ± 5.2 ^b^	**<0.001**
	**Triceps skin fold thickness (mm)**	15.68 ± 7.8	12.12 ± 5.6^b^	15.07 ± 7.1^b^	20.41± 8.2^b^	**<0.001**
	**MAC (cm)**	27.81 ± 4.9	25.42 ± 4.4^b^	27.59 ± 3.7^b^	30.89± 4.3^b^	**<0.001**
	**CC (cm)**	32.86 ± 5.3	29.97 ± 4.1^b^	32.45 ± 3.6^b^	36.66 ± 4.9^b^	**<0.001**

Laboratory and anthropometric data are presented as mean ± SD or median (interquartile range)

*BMI* Body mass index, *MAC* Mid-arm circumstances, *CC* Calf circumference

Thresholds of nutritional risk severity by the Geriatric Nutritional Risk Index were:

<92, high risk; 92 to 98, low risk; >98, no risk

Bold values indicate significant values

^a^*P*‐value according to ANOVA, Kruskal-Wallis, and Pearson’s Chi-square tests

^b^Significantly different from the other groups by post-hoc comparison

The GNRI score of all patients ranged from 63.00 to 147.90, with a mean value of 95.07 ± 13.63. The prevalence of high, low, and no nutritional risk as measured by GNRI was 45.5% (95% CI, 40%–51%), 18% (95% CI, 13.9%–22.5%), and 36.5% (95% CI, 31.3%–41.9%), respectively**.**

There was a statistically significant difference in the development of bed sores, HAIs, hospital-acquired pneumonia, and urinary tract infection among different nutritional risk groups (p<0.05), with incidence rates worsening as the nutritional risk increased. Patients in the high-risk group had a significantly longer hospital LOS, as median hospital days significantly increased in patients with no, low, and high risk from 8 to 10 and 12 days, respectively. Additionally, hospital mortality significantly increased as nutritional risk increased as the incidence of hospital deaths among patients of the high-risk group was 15.1% (95% CI, 9.8%–21.8%) compared to 3.3% (95% CI, 0.9%–8.1%) mortality rate in no-risk group. Similarly, the incidence rate of deterioration in the medical condition and transfer rate to ICU was significantly higher 18.4% (95% CI, 12.6%–25.5%) among the high-risk group compared to low, no risk (10.0%, 4.1%) respectively. Also, patients at high nutritional risk were less frequently discharged to home compared to patients at no risk (61.2% and 86.1%) respectively (Table 2).

**Table 2:** Association between GNRI and clinical outcomes occurred at the hospital and during follow-up

**Health Complications**		**Total patients (*n*=334)**	**High nutritional risk (*n*=152)**	**Low nutritional risk (*n*=60)**	**No nutritional risk (*n*=122)**	***P*-value**^**a**^
		**No. (%)**				
**During hospitalization**						
**Bed sores**		31 (9.3)	21 (13.8)	4 (6.7)	6 (4.9)	**0.031**
**Healthcare-associated Infections (HAIs)**		102 (30.5)	64 (42.1)	18 (30.0)	20 (16.4)	**<0.001**
**• Hospital-acquired Pneumonia**		27 (8.1)	17 (11.2)	6 (10.0)	4 (3.3)	**0.048**
**• Surgical Wound Infection**		10 (3.0)	8 (5.3)	1 (1.7)	1 (0.8)	0.079
**• Urinary tract infection**		30 (9.0)	20 (13.2)	3 (5.0)	7 (5.7)	**0.050**
**• Catheter infection**		1 (0.3)	1 (0.7)	0 (0.0)	0 (0.0)	1.000
**• Blood infection**		2 (0.6)	2 (1.3)	0 (0.0)	0 (0.0)	0.667
**• Oral infection**		1 (0.3)	1 (0.7)	0 (0.0)	0 (0.0)	1.000
**• Hospital-acquired COVID-19 infection**		50 (15.0)	27 (17.8)	12 (20.0)	11 (9.0)	0.063
**Hospital LOS**	**Median (IQR)**	10 (8)	12 (12) ^b^	10 (6)	8 (8)	**0.001**
**Outcome at Discharge**	**Death**	30 (9.0)	23 (15.1)	3 (5.0)	4 (3.3)	**<0.001**
	**Transfer to another unit**	17 (5.0)	8 (5.3)	1 (1.7)	8 (6.6)	
	**transfer to ICU**	39 (11.7)	28 (18.4)	6 (10.0)	5 (4.1)	
	**to home**	248 (74.3)	93 (61.2)	50 (83.3)	105 (86.1)	
**During follow up after discharge**						
**Medical condition improved after discharge (*n*=304)**	**Yes**	71 (23.4)	13 (10.1)	11 (19.3)	47 (39.8)	**<0.001**
	**partially**	61 (20.1)	19 (14.7)	14 (24.6)	28 (23.7)	
	**No**	118 (38.8)	69 (53.5)	21 (36.8)	28 (23.7)	
	**loss of follow up**	54 (17.8)	28 (21.7)	11 (19.3)	15 (12.7)	
**New medical conditions come up (*n*=250) **^**c**^		132 (52.8)	75 (74.3)	27 (58.7)	30 (29.1)	**<0.001**
**Readmission to hospital (*n*=250)**^**c**^		55 (22.0)	24 (23.8)	12 (26.1)	19 (18.4)	0.449
**Death during follow up (*n*=250) **^**c**^		42 (16.8)	21 (20.8)	10 (21.7)	11 (10.7)	0.095
**Overall mortality (*n*=280)**^**d**^		72 (25.7)	44 (35.5)	13 (26.5)	15 (14.0)	**0.001**

*LOS* Length of stay

Bold values indicate significant values

^a^*P*‐value according to Chi‐square, Fisher exact, and Kruskal‐Wallis tests

^b^Significantly different from the other groups by Mann-Whitney test with Bonferroni correction

^c^The percentages were calculated after the removal of patients who died at the hospital and those who lost to follow up

^d^The percentages were calculated after removing patients who lost to follow up

During the three-month follow-up period, there were 54 patients lost to follow-up. Among the high-risk group (53.5%) of patients reported no improvement in their medical condition compared to (23.7%) in the no-risk group. The appearance of new medical conditions was significantly reported more frequently among the high-risk group compared to no-risk (74.3% and 29.1%) respectively. These differences were statistically significant. Patients in the high-nutritional risk group had higher 90-day hospital readmission and 90-day mortality rates compared to those in the no-risk group. However, the difference was statistically insignificant (*p* > 0.05) (Table 2).

Patients with nutritional risk had increased risk of ICU transferal (Relative Risk (RR): 3.91; 95% CI, 1.57–9.74), hospital mortality (RR: 3.74; 95% CI, 1.33–10.46), and overall mortality (RR: 2. 18; 95% CI, 1.29–3.69) (Table 3).

**Table 3:** Relative Risk for some adverse outcomes

**Variables**	**Nutritional risk (*n*=212)**	**No nutritional risk (*n*=122)**	***P*-value**^**a**^	**Relative Risk**	**95% C.I.**
	**No. (%)**				
**Bed sores**	25 (11.8)	6 (4.9)	**0.037**	2.39	1.01 – 5.68
**HAIs**	82 (38.7)	20 (16.4)	**<0.001**	2.35	1.52 – 3.64
**Transfer to ICU after a period of hospitalization**	34 (16)	5 (4.1)	**0.001**	3.91	1.57 – 9.74
**Hospital mortality**	26 (12.3)	4 (3.3)	**0.006**	3.74	1.33 – 10.46
**Overall mortality**	57(26.9)	15 (12.3)	**0.002**	2.18	1.29 – 3.69

*HAIs* Healthcare-Associated Infections, *CI* Confidence Interval

Bold values indicate significant values

^a^*P*‐value according to Chi‐square test

In a linear regression where age, body mass index, and presence of comorbidities were adjusted, the nutritional risk was significantly associated with prolonged hospital LOS. On average, patients with a high nutritional risk stayed in the hospital for 3.6 days longer than those with no nutritional risk (Table 4).

**Table 4:** Predictors of Hospital length of stay using multiple linear regression

**Model**	**Unstandardized Coefficients**		**Standardized Coefficients**	**t**	**Sig.**	**95% C.I. for B**
	**B**	**Std. Error**	**Beta**			
**Age**	-0.019	0.054	-0.019	-0.362	0.718	-0.12 – 0.08
**BMI**	0.191	0.106	0.115	1.801	0.073	-0.01 – 0.39
**Presence of comorbidities**	0.853	2.766	0.016	0.308	0.758	-4.58 – 6.29
**High nutritional risk**	3.611	1.209	0.220	2.986	**0.003**	1.23 – 5.99
**Low nutritional risk**	0.043	1.301	0.002	0.033	0.973	-2.51 – 2.60
**Bed sores developed in the hospital**	3.474	1.484	0.134	2.551	**0.011**	0.86 – 6.70
**HAIs**	4.132	0.956	0.232	4.322	**<0.001**	2.25 – 6.01

Bold values indicate significant values

*BMI* Body Mass Index, *HAIs* Healthcare-Associated Infections, *CI* Confidence Interval

Geriatric Nutritional Risk Index threshold values: <92, high risk; 92 to 98, low risk.

In multivariable logistic regression and after controlling for confounding variables, the high nutritional risk was an independent predictor of bed sores developed at the hospital (AOR: 4.89; 95% CI, 1.37–17.45), HAIs (AOR: 3.18; 95% CI, 1.48–6.83), non-improvement in the medical status after discharge (AOR: 3.55; 95% CI, 1.69–7.47), and appearance of new medical problems during follow-up (AOR: 4.99; 95% CI, 2.59–9.61) (Table 5).

**Table 5:** Multivariable logistic regression Analysis of GNRI With different patient outcomes

**Outcomes**	**High nutritional risk **^**a**^			**Low nutritional risk**		
	**AOR **^**b**^	**95% C.I.**	***P* value**	**AOR**	**95% C.I.**	***P* value**
**Bed sores**	4.89	1.37 – 17.45	**0.014**	2.22	0.52 – 9.37	0.275
**Healthcare-associated infections**	3.18	1.48 – 6.83	**0.003**	2.23	0.99 – 5.09	**0.051**
**Hospital mortality**	4.41	1.04 – 18.59	**0.043**	1.69	0.31– 9.16	0.539
**No improvement of the medical status after discharge**	3.55	1.69 – 7.47	**0.001**	2.36	1.03 – 5.42	**0.042**
**New medical conditions come up during follow up**	4.99	2.59 – 9.61	**<0.001**	3.28	1.52 – 7.08	**0.002**
**Hospital readmission**	1.19	0.57 – 2.49	0.639	1.42	0.61 – 3.33	0.411
**90-day mortality after discharge**	1.56	0.65 – 3.70	0.313	2.47	0.92 – 6.63	0.072

Bold values indicate significant values

*AOR* Adjusted Odds Ratio, *CI* Confidence Interval, *GNRI* Geriatric Nutritional Risk Index

^a^Geriatric Nutritional Risk Index threshold values: <92, high risk; 92 to 98, low risk; >98, no risk (reference category)

^b^All the models were adjusted for age, body mass index, presence of comorbidities, and hospital LOS

**Fig. 1:**
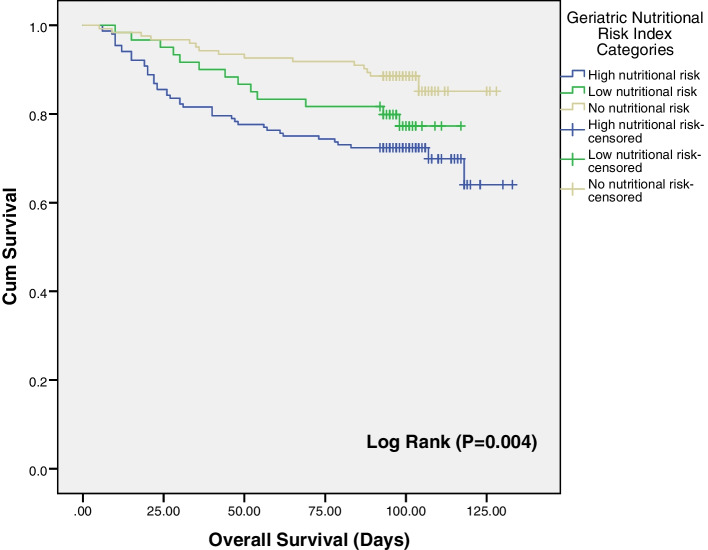
Kaplan-Meier analysis of overall survival according to GNRI

In survival analysis, Kaplan-Meier curves for all-cause death showed that the overall survival rate was significantly worse in the high-risk group than in the no-risk group, and lower mean survival days were observed in the high-risk group compared to the no-risk (103 and 117 days) respectively. The difference between survival rates among nutritional risk groups was tested by log-rank test and was statistically significant (*P* = 0.004) (Fig. 1).

On Cox hazard regression analysis, patients in the high nutritional risk group had a higher risk of overall mortality compared to those in the no-risk groups (AHR: 2.06; 95% CI: 1.10–3.85, *P*=0.024). Patients with prolonged hospital LOS had an increased risk of overall mortality (AHR: 1.03; 95% CI: 1.01–1.06, *P*=0.004). (Table 6).

**Table 6:** Predictors of overall mortality according to Cox proportional hazard regression

**Variables in the model**	**B**	**S.E.**	**Sig.**	**AHR**	**95% C.I. for HR**
**Age**	0.021	0.015	0.168	1.02	0.99 – 1.05
**Hospital LOS**	0.037	0.013	**0.004**	1.03	1.01 – 1.06
**GNRI **^**a**^					
High nutritional risk	0.723	0.320	**0.024**	2.06	1.10 – 3.85
Low nutritional risk	0.655	0.388	0.091	1.92	0.90 – 4.11
**bed sores developed in the hospital**	0.598	0.322	0.064	1.81	0.96 – 3.42
**HAIs**	-0.081	0.277	0.770	0.922	0.53 – 1.58

Bold values indicate significant values

*AHR* Adjusted Hazardous Ratio, *CI* Confidence Interval, *LOS* Length of Stay, *GNRI* Geriatric Nutritional Risk Index, *HAIs* Healthcare-Associated Infections

^a^Geriatric Nutritional Risk Index threshold values: <92, high risk; 92 to 98, low risk; >98, no risk (reference category)

## Discussion

Malnutrition is a major geriatric condition that is prevalent among elderly hospitalized patients. It remains underreported, often underdiagnosed, and considered to be one of the contributing factors for worse health outcomes and increased morbidity and mortality [26]. The GNRI's benefits include being a quick and objective nutrition screening tool that requires little involvement from patients and being dependent on current body weight, which eliminates bias related to past unintentional weight loss investigations [23].

This study directly assessed the capability of the GNRI score as a prognostic index for the prediction of nutrition-related morbidity and mortality in an acute care setting in Cairo, Egypt. In this study, the prevalence of high nutritional risk was (45.5%) which is higher than that reported by an old cohort study conducted in the same hospital over a decade ago which revealed that the prevalence of high nutritional risk as assessed by GNRI was (41.2%) [9]. The present higher rate of high nutritional risk denotes that almost half of the admitted patients are at risk of nutrition-related complications including mortality. This also implies that malnutrition status is on the rise among elderly patients admitted to hospitals in Egypt.

Similarly, previous studies nearly agreed with the current study where the prevalence of high risk was (49.7% and 48.4%) respectively [27, 28]. This observation strengthens public health concerns regarding the nutritional risk of health complications in the elderly population.

The present study showed that the nutritional risk significantly increased with advancing patient age. This coincides with a prospective multicenter cohort study in an acute hospital setting conducted in Italy [29]. This relation between age and nutritional risk is expected given that malnutrition and ageing are linked in the elderly. And the fact that many changes related to ageing such as anorexia, decreased taste and smell, and a decrease in gastric acid secretion which affects the absorption of multiple nutrients can cause malnutrition.

There was a statistically significant difference between the preadmission status and nutritional risk as among the high-risk group, more than half (52.6%) were in the ICU prior to ward admission. The metabolic reaction to serious illness may provide an explanation for this finding. The body shifts to a hypercatabolic state during critical illness conditions, as the patient suffers from a high degree of stress and inflammation, which causes the body to catabolize more proteins and other substances to meet the patient's increased energy demands and maintain physiological functions [30].

Regarding the anthropometric parameters, the present study revealed that increasing nutritional risk was associated with more depleted nutritional parameters. Significant differences were detected in the parameters of skinfold thickness, MAC, and CC in the GNRI groups. In addition, BMI was detected in high, low, and no nutritional risk groups (23.5, 26.0, and 29.9) respectively. This result was further agreed with other studies that found that the high nutritional risk group had a BMI and serum albumin lower than the other groups [29, 31]. These results suggested that simple and low-cost parameters such as the anthropometric measures are probably valid parameters for estimating nutritional status in elderly hospitalized inpatients.

The utilization of both albumin and weight in the index minimizes different confounding variables such as inflammation and hydration status. According to a Japanese study, the GNRI was more accurate at predicting morbidity and mortality than either the BMI or albumin alone [32].

Regarding the adverse clinical outcomes, as the level of nutritional risk increased, the incidence of complications increased. In the present study, the incidence of HAIs in high, low, and no nutritional risk was (42.1%, 30%, and 16.4%) respectively. A similar incidence rate was reported in a previous study mentioned that the incidence of HAIs in high, low, and no nutritional risk was (41.7%, 25.5%, and 20.6%) respectively [28]. This is also in accordance with another study reported that severe malnutrition defined by GNRI is associated with a higher risk of complications [18]. So, GNRI quantifies the severity of malnutrition and its impact on individual complications.

The present study also found that high and low nutritional risk were significant independent predictors for HAIs complications. This result was further agreed with a study found that high nutritional risk was an independent risk factor of postoperative pneumonia, surgical site infection, sepsis, and urinary tract infection [33]. In the same context, the present study illustrated that bed sores developed at the hospital were significantly associated with high nutritional risk. This finding was supported by a study reported that GNRI was detected as a significant independent predictor for bed sores complications [23].

The association between malnutrition and hospital LOS is well‐established. One previous study suggested that the risk of malnutrition, as assessed using the GNRI, contributed to prolonged LOS in elderly patients [29]. The results of the present study were consistent with that previous finding as they showed a significant association between prolonged LOS and nutritional risk, the median hospital days significantly increased in patients with no, low, and high risk from 8 to 10 and 12 days, respectively. This issue is of special interest as clinical decision-making concerning nutritional screening and therapeutic interventions is often driven by economic factors [34].

In this study, the incidence of hospital mortality among patients of the high-risk group was (15.1%) this observation agrees with a study conducted on elderly inpatients admitted to a teaching hospital in Seoul, Korea which reported that (21.7%) of high nutritional risk patients died in the hospital within 28 days [35]. The difference in hospital readmission rate between GNRI groups, as assessed in this study, didn't quite reach statistical significance. One potential reason is that the cause of rehospitalization is multifactorial and is related not only to the severity of malnutrition but also to patient self-care and socioenvironmental factors. In this study, most patients who were readmitted to the hospital were because of different factors not related to malnutrition as undergoing an endoscope (previously scheduled at discharge).

There was a much lower overall survival rate in cases with high nutritional risk compared to the normal group and the difference is highly statistically significant (*P* = 0.004). Consistency to this result, a study conducted on elderly patients admitted to critical care units in Boston, USA and found that the 90-day survival was significantly lower in the group with nutrition risk (GNRI ≤ 98) compared with the no-risk group (GNRI > 98) [36].

Although an old cohort study which was conducted in the same hospital a decade ago reported the validity and simplicity of the GNRI tool for prediction of nutrition-related morbidity and mortality complications in elderly hospitalized patients [9], yet this nutritional screening tool is not applied in the geriatric hospital or considered as a screening tool.

The findings of the present study indicate the need for a reliable and simple index for the early detection of the risk of malnutrition in Elderly hospitalized patients all over Egypt. And, with fast detection comes the need for close and thorough follow-up from dietitians in this high-risk group to lower mortality among these categories. So, there is the utmost need for the application of this geriatric nutritional screening tool in Egyptian hospitals.

### Limitations of this study

Single time point measurement of the GNRI at admission was used for the analyses. This single measurement may have failed to detect the intraindividual variability in the albumin level over time and may result in the misclassification of the patients into different GNRI level categories. It is not always easy to measure the current weight of acute bedridden patients. Another limitation is the COVID-19 pandemic because it forced the geriatric hospital to close and become an isolation facility for confirmed COVID-19 cases. This made it difficult to collect data for a while. Finally, this was a single-center study, the results may not be generalizable to different clinical settings.

## Conclusions

In conclusion, GNRI is a simple and objective nutritional screening method that could be used to give warning on short-term and long-term risks of morbidity and mortality. Nutritional risk, as defined by GNRI, is an independent predictor of multiple health adverse outcomes such as bed sores developed during hospitalization, HAIs, and prolonged hospital LOS. Therefore, using GNRI to assess elderly patients' nutritional status may help to identify patients who are at high risk of adverse outcomes more quickly and allow for early intervention with appropriate and timely nutritional care management to mitigate the risk of morbidity, improve clinical outcomes, and reduce the costs of healthcare.

## Data Availability

The datasets used and/or analyzed during the current study are available from the corresponding author upon reasonable request.

## Abbreviations

ASU: Ain Shams University
GNRI: Geriatric Nutritional Risk Index
HAIs: Healthcare-Associated Infections
LOS: Length of Stay
CI: Confidence Interval
AOR: Adjusted Odds Ratio
ICU: Intensive Care Unit
MIS: Malnutrition Inflammation Score
SGA: Subjective Global Assessment
MNA-SF: Mini Nutritional Assessment–Short Form
MUST: Malnutrition Universal Screening Tool
MST: Malnutrition Screening Tool
NRS-2002: Nutritional Risk Screening 2002
ESPEN: European Society of Clinical Nutrition and Metabolism
MNA: Mini Nutritional Assessment
BMI: Body Mass Index
MAC: Mid-Arm Circumference
CC: Calf Circumference
EH: Estimated Height
KH: Knee-Heel
SD: Standard Deviation
IQR: Inter Quartile Range
ANOVA: Analysis of Variance
RR: Relative Risk
OS: Overall Survival
AHRs: Adjusted Hazard Ratios
